# A Data Enhancement Algorithm for DDoS Attacks Using IoT

**DOI:** 10.3390/s23177496

**Published:** 2023-08-29

**Authors:** Haibin Lv, Yanhui Du, Xing Zhou, Wenkai Ni, Xingbang Ma

**Affiliations:** College of Information and Cyber Security, People’s Public Security University of China, Beijing 100038, China; 18259568358@163.com (H.L.); 2021212347@stu.ppsuc.edu.cn (X.Z.); 2021211449@stu.ppsuc.edu.cn (W.N.); 2021212361@stu.ppsuc.edu.cn (X.M.)

**Keywords:** internet of things, imbalanced classification, oversampling, normal distribution

## Abstract

With the rapid development of the Internet of Things (IoT), the frequency of attackers using botnets to control IoT devices in order to perform distributed denial-of-service attacks (DDoS) and other cyber attacks on the internet has significantly increased. In the actual attack process, the small percentage of attack packets in IoT leads to low accuracy of intrusion detection. Based on this problem, the paper proposes an oversampling algorithm, KG-SMOTE, based on Gaussian distribution and K-means clustering, which inserts synthetic samples through Gaussian probability distribution, extends the clustering nodes in minority class samples in the same proportion, increases the density of minority class samples, and improves the amount of minority class sample data in order to provide data support for IoT-based DDoS attack detection. Experiments show that the balanced dataset generated by this method effectively improves the intrusion detection accuracy in each category and effectively solves the data imbalance problem.

## 1. Introduction

According to the IoT OS Security 2022 White Paper [1], the global number of IoT connections is continuously increasing, showing a high growth trend. In 2020, there were 13.1 billion global IoT connections, and this number is projected to reach 24.6 billion IoT devices by 2025. With more than 30% of the global IoT connections, our current growth trajectory is promising.

The proliferation of IoT devices has also led to a significant rise in cyber attacks, particularly Distributed Denial of Service (DDoS) attacks perpetrated by attackers leveraging IoT devices. One notable example is the Mirai attack in 2016, which resulted in the infection of millions of IoT devices and the launch of DDoS attacks against DYNs, causing substantial economic losses. The CNCERT Internet Security Threat Report reveals that, as of 2022, over 4.78 million botnet agents were utilized in DDoS attacks, some of which were IoT devices [2]. The use of IoT devices to carry out DDoS attacks has become the prevailing method of attack. These attacks are characterized by their large scale, low local traffic impact, and diverse attack traffic protocols. Consequently, detecting such attack traffic poses a significant challenge for intrusion detection systems, leading to a high false alarm rate. Overall, the increasing prevalence of IoT-enabled DDoS attacks presents a critical challenge for intrusion detection systems due to the difficulty in accurately detecting and mitigating this form of attack.

Traditional classification models assume a balanced sample distribution when addressing classification problems [3]. However, in reality, intrusion detection systems use datasets that are imbalanced, meaning that some classes have significantly fewer samples than others. This poses a challenge, as standard classifiers tend to prioritize optimizing the overall classification error, which may sacrifice the accuracy of minority classes [4]. To tackle this problem, scholars have proposed various strategies, broadly categorized into three approaches: data-level, algorithm-level, and integrated learning approaches [5].

The data-level approach involves modifying the original dataset through data preprocessing and resampling in the sample feature space in order to obtain a balanced dataset. The algorithm-level approach focuses on enhancing or integrating classification algorithms in order to improve data differentiation and classification accuracy. The integrated learning approach combines data-level and algorithm-level methods with integrated learning techniques to adapt to the requirements of unbalanced data classification and enhance the learning of minority class samples.

In this paper, the focus is on data-level methods as they are implemented during the data preprocessing stage and can be applied generally. Specific strategies are used to increase the density and diversity of minority class samples without altering the sample distribution, aiming to reduce or solve the issue of imbalanced data. The methodology includes undersampling techniques [6], oversampling techniques, and combined under- and oversampling techniques [7].

Undersampling techniques involve balancing the dataset by removing some majority samples in order to achieve balance. However, removing majority samples may cause the classification algorithm to lose important information about the majority classes, leading to a decrease in intrusion detection accuracy. Therefore, this paper focuses on the oversampling technique to address the data imbalance problem [8]. The basic idea behind oversampling is to add minority samples to the dataset in order to achieve a balanced sample distribution.

This paper proposes a novel oversampling method for unbalanced data called KG-SMOTE, which is based on Gaussian distribution and K-means clustering. The main objective of this method is to improve the prediction accuracy of minority categories, particularly the minority categories with limited sample sizes. The proposed approach aims to achieve balance among all categories by adjusting the distribution of the minority categories, ensuring that their sample sizes are not significantly different from the majority categories. To achieve this, the KG-SMOTE algorithm is employed to generate new samples for the minority category.

The motivation behind developing the KG-SMOTE method is to address the challenge of extremely unbalanced datasets. The utilization of the K-means clustering algorithm allows for the division of the minority class into distinct clusters with similar attributes. This approach enables the extraction of additional information from each cluster, revealing hidden patterns or groupings in the data. Moreover, it helps to reduce the generation of noisy samples. The adoption of Gaussian distribution is another key aspect of the KG-SMOTE method. This choice ensures that the synthetic data generated maintain similar probability distributions to the original minority data. It also addresses the issue of overfitting that can arise from an excessive concentration of synthetic samples produced by traditional SMOTE in extremely unbalanced datasets.

The remaining sections of this paper are structured as follows. Section 2 presents a review of recent approaches to addressing the class imbalance problem, with a focus on oversampling techniques. In Section 3, a brief background is provided on existing oversampling methods along with an overview of classification algorithms. The proposed method is presented in detail in Section 4. Section 5 presents and discusses the experimental results obtained from applying the proposed method. Finally, Section 6 summarizes the key findings of the study and provides recommendations for future research.

## 2. Related Works

There are different approaches in the literature for dealing with the class imbalance problem. This section reviews recent methods and techniques used for balancing data. Table 1 presents a comprehensive summary of recent advancements in various oversampling techniques.

Random Oversampling (ROS) is the most straightforward oversampling technique. Since the ROS method reaches the equilibrium point by replicating a few samples in order to expand the dataset, it does not generate new samples. Therefore, it is prone to overfitting problems during the training of classification algorithm models and has some limitations in practical applications.

Chawla [9] proposed a synthetic minority oversampling technique (SMOTE). This technique finds K neighbor nodes of minority class sample points in the sample feature space, randomly selects a neighbor node, and synthesizes a new sample between the sample point and its neighbor node. Still, this method increases the possibility of overlap and noise between classes.

**Table 1:** The current development status of oversampling techniques.

Work	Year	Algorithms	Dataset	Results
[10]	2022	GMM-SMOTE	UCI	On average, the AUC value has been improved by 6.09%.
[11]	2017	K-means SMOTE	UCI	To a certain extent, has addressed the issue of noise and has alleviated intra-class imbalance.
[12]	2023	RUCSMOTE	KEEL	AUC and GM have generally increased by 2 to 7 percentage points.
[13]	2021	GSMOTEBoost	KEEL	AUC and GM have generally improved by 1 to 3 percentage points.
[14]	2022	HDP-SMOTE	NSL-KDD UNSW-NB15	F1 score and GM have generally shown an improvement of 1 to 6 percentage points.
[15]	2019	KDE	[15]	F1 score and GM have demonstrated a general enhancement ranging from 0.6 to 7 percentage points.
[16]	2011	NDO	UCI	The computational complexity has been reduced.
[17]	2021	GK-Means	[17]	The F1-score and accuracy have exhibited a general improvement ranging from 1 to 6 percentage points.
[18]	2019	PDE-SMOTE	UCL KEEL	The F1-score and GM have experienced a general enhancement ranging from 1 to 3 percentage points.
[19]	2020	SGM	UNSW-NB15	The detection rate has reached 99.74% in binary classification and 96.54% in multi-class classification.

Yehui et al. [10] proposed a SMOTE oversampling technique based on Gaussian mixture model clustering. The GMM algorithm is first used to cluster several class sample sets. Redundant samples that overlap with the cluster centroids are removed, and, finally, SMOTE oversampling is performed according to different clusters in order to make the data balanced. The three algorithms of RF, SMOTE+RF, and GMM-SMOTE+RF are used to experiment with the classification effect on six sets of UCI standard open datasets. This method can handle the intra-class imbalance problem better, but the classification effect is poor for highly imbalanced datasets.

Last F [11] used the K-mean clustering algorithm and SMOTE technique for oversampling to cope with the imbalance problem. The method proposed in this paper follows three main steps: clustering, filtering, and oversampling. Clustering uses the K-means algorithm to divide the dataset into groups based on the value of K. Then, filtering is used to select clusters for oversampling based on a small number of classes of samples. Finally, the SMOTE technique is applied for oversampling in order to balance the dataset. This method is a good solution to the problem of generating noisy samples and class overlap, but there is no clear method for the selection of the number of clusters.

Zhu Shen et al. [12] proposed the RUCSMOTE algorithm based on the K-means SMOTE technique, incorporating random undersampling techniques. The paper proposed to take the cluster center point in each cluster, the nearest point to the cluster center, and a random point in the cluster on the three points to create a triangle, and take a random point on its vertical line for interpolation to solve the synthetic sample point overfitting problem, which is only applicable to datasets with high imbalance ratios. The advantage of the algorithm is not obvious when the number of samples is small and the imbalance ratio is low.

Zhang Z et al. [13] proposed an algorithm to construct the unbalanced learning model under the Boosting integration framework. To improve the robustness of the classification system, a Gaussian process SMOTE-based oversampling technique is used to increase the diversity of training samples for the base classifiers in order to improve the differences between the base classifiers. To verify the effectiveness of the algorithm, the commonly used algorithms dealing with unbalanced classification problems are used as a comparison method, and the algorithm is tested with 20 standard datasets in the KEEL database. The G-mean, F-measure, and AUC are used as the evaluation indexes of the algorithm, and the results of the experiments are analyzed by using statistical tests. The experimental results show that the proposed GSMOTEBoost has significant advantages over other algorithms.

Jiang Zetao et al. [14] proposed a data generation scheme based on a combination of dense and sparse. The sparse generation scheme is based on the highest density points and the average intra-class distance in order to reduce the original minority class sparse clustering range to the minority class denser region, increasing the possibility of the minority class samples being oversampled. The dense scheme is a radial SMOTE method in the non-dense region. In the non-dense region, only the dense solution uses the radial SMOTE method in the non-dense area, so that only the highest density points of the target class samples, and the sample points in the non-dense area, are focused on the non-dense area, thus avoiding the sample overlap problem. However, this method still has the problem of noisy samples.

Kamalov et al. [15] proposed a technique called kernel density estimation (KDE) for oversampling unbalanced datasets. The authors proposed that Gaussian functions are used as kernels in KDE. Experimental results show that this method can provide higher performance compared to other related methods. SVM, KNN, and MLP algorithms are used to evaluate the classification performance on 14 different datasets. The classification performance is better for highly unbalanced datasets, but it is prone to generating noisy samples.

Zhang H et al. [16] proposed a normal-distribution-based oversampling method to balance the number of instances belonging to different classes in the dataset. The balanced training data are used to train an unbiased classifier for the original dataset. Under certain conditions, the proposed oversampling method produces samples with expected mean and variance similar to the original minority class data. Since the method attempts to generate synthetic data with similar probability distributions as the original data and extends the class boundaries of the minority class, it may improve the classification performance of the minority class. Experimental results show that the method outperforms other methods in most cases on benchmark datasets implementing several classical classification algorithms.

Hassan et al. [17] proposed a simple oversampling method based on multivariate Gaussian distribution and K-means clustering called GK-Means. The method aims to avoid generating noise and to control the imbalance between minority and majority classes and within the minority class. Various experiments were conducted with six classifiers and four oversampling methods. The experimental results on different unbalanced datasets show that the proposed GK-Means oversampling replaces the other oversampling methods and improves the classification performance of F1-score and accuracy.

Li Tao et al. [18] proposed an improved algorithm based on probability density estimation. Firstly, it is assumed that all the samples obey Gaussian mixture distribution, the probability density of each sample is measured by the Gaussian mixture model, and the filtering of noise information is achieved by comparing the rankings of the probability densities of the samples within and between the classes. Secondly, the probability densities are recalculated on the filtered samples, which are classified into three categories according to their characteristics: boundary samples, safe samples, and outlier samples. Finally, for the above three types of samples, different strategies are adopted for SMOTE sampling.

Zhang H et al. [19] proposed a new large-scale dataset class imbalance processing technique, SGM, which combines SMOTE and undersampling techniques for clustering based on Gaussian mixture models. They designed a stream-based intrusion detection model, SGM-CNN, which integrates imbalanced class processing with convolutional neural networks, and they investigated the effects of different numbers of convolutional kernels and different learning rates on the model performance.

The aforementioned work has made significant contributions to addressing class imbalance in various domains, such as network datasets, financial data, and medical data. However, most of these methods focus on algorithmic improvements using existing datasets and do not specifically target algorithmic enhancements for DDoS attack scenarios in the context of the Internet of Things.

This paper aims to fill this gap by proposing the KG-SMOTE oversampling algorithm, designed specifically for IoT devices launching DDoS attacks against internet servers and clients. Taking into consideration the characteristics of IoT traffic and highly imbalanced datasets, this method greatly improves the detection effectiveness for DDoS attacks initiated by different virus protocols.

## 3. Background

This section reviews some existing well-known resampling methods.

### 3.1. SMOTE

SMOTE is a widely used oversampling method for addressing the issue of imbalanced datasets with minority class samples. This algorithm aims balance the sample distribution among different classes by generating synthetic samples.

The SMOTE algorithm follows a series of steps. First, it iterates through all minority class samples and calculates the Euclidean distance between each minority class sample and other minority class samples. Next, for each minority class sample, the algorithm randomly selects a sample from its k-nearest neighbors. Using the distance information between these samples, new synthetic samples are generated.

By adding these synthetic samples to the original dataset, the number of minority class samples is increased to match that of the majority class samples. This helps to alleviate the class imbalance problem. The formula for synthesizing samples in SMOTE is as follows:

(1) $$x_{new}=x_{i}+\varepsilon \cdot (x_{i}-x_{j})$$

where $x_{new}$ is the synthesized minority class sample, $x_{i}$ is the i minority class sample, $x_{j}$ is the j nearest neighbor sample of the i minority class sample, and ε is a random number between [0, 1].

The SMOTE method balances the class distribution in the dataset by synthesizing new minority class samples in order to increase the number of minority class samples. This reduces the classifier’s over-preference for majority class samples and improves the recognition of minority classes.

### 3.2. K-Means SMOTE

The K-Means SMOTE algorithm incorporates the k-means clustering step the traditional SMOTE algorithm in order to address the data imbalance issue. This algorithm first clusters the samples using k-means and calculates the Euclidean distance between them. It then selects the centroid of each cluster for SMOTE processing, generating new synthetic samples.

By including the k-means clustering step, the K-Means SMOTE algorithm effectively preserves the features of the minority class samples. This approach reduces the generation of noisy points and outliers compared to the traditional SMOTE algorithm. As a result, the performance of the classifier is enhanced.

### 3.3. Gaussian Probability Distribution

The Gaussian probability distribution, also known as the normal distribution, is a continuous probability distribution that is commonly observed in everyday life. It is considered the most important distribution, as it applies to a wide range of events. The principle behind the Gaussian distribution is based on the central limit theorem, which states that the sampling distribution of means tends to converge to a normal distribution, regardless of the true underlying distribution.

When applying the Gaussian probability distribution for data analysis, there are typically five steps involved. First, relevant data related to the subject of study are collected. Next, the data are pre-processed to ensure accuracy and consistency by removing outliers or other necessary steps. The parameters of the Gaussian distribution, such as the mean and standard deviation, are then estimated using methods such as maximum likelihood estimation. These estimated parameters are used to obtain the Gaussian distribution curve through distribution fitting methods. Finally, the data are analyzed and inferences are made based on the properties of the Gaussian distribution, such as calculating probabilities and determining confidence intervals.

The probability density function of the Gaussian distribution is represented by the formula shown in [20]:

(2) $$f\left(x\right)=\frac{1}{\sigma \sqrt{2\pi }}e^{-\frac{{\left(x-\mu \right)}^{2}}{2\sigma^{2}}}$$

where x is the random variable, μ and $\sigma^{2}$ are the mean and variance parameters of the distribution, and σ is the standard deviation. The mean μ and standard deviation σ of the sample are defined as follows:

(3) $$\mu =\frac{1}{n}\sum_{i=1}^{n}x_{i}$$

(4) $$\sigma =\sqrt{\frac{1}{n-1}\overset{n}{\underset{i=1}{\sum }}{\left(x_{i}-\bar{x}\right)}^{2}}$$

## 4. KG-SMOTE

To address the imbalance in the dataset, it is necessary to generate a large number of synthetic data samples. However, this approach can result in excessive overlap of synthetic data, leading to duplicated features in the minority sample set. This, in turn, can cause the classification algorithm model to overfit the data. Overfitting occurs when the model learns too much from a few classes of samples, leading to a decrease in detection accuracy. Moreover, due to the characteristics of IoT DDoS attacks, each device issues attack protocols and formats that differ from one another. This further increases the data imbalance ratio, which negatively impacts the accuracy of detecting the same attack type.

To address these challenges, this study proposes an improved oversampling algorithm called KG-SMOTE, which is based on K-Means clustering and Gaussian probability distribution. The KG-SMOTE algorithm enhances the intrusion detection of imbalanced IoT attack data from a data-level perspective.

The KG-SMOTE algorithm first employs the K-Means clustering algorithm to divide the minority classes into different clusters with similar attributes. This step helps reduce interference from noise and anomalous samples. Secondly, the algorithm utilizes a Gaussian probability distribution to generate synthetic data with a similar probability distribution as the original minority class data. This approach improves the quality of the generated samples in datasets with high imbalance ratios, reduces the generation of redundant and noisy samples, and mitigates the occurrence of overfitting in the classification model.

The KG-SMOTE algorithm is based on the K-Means SMOTE algorithm, which combines a Gaussian distribution to generate new samples during synthetic data generation. The K-Means algorithm is used to cluster the minority classes in the dataset, calculate the number of minority class samples in each cluster, and increase the density of minority class nodes in equal proportion. The number of synthetic samples is determined by treating each attribute of the training data as a random variable, and all attributes are independent of each other. The given m attributes, denoted as $a_{1}$, $a_{2},\ldots ,a_{m}$, indicate that there are m random variables. Based on a few classes of samples in each cluster, we compute the expected value and variance of each random variable. The mean and standard variance of $a_{i}$ are denoted as $\mu_{i}$ and $\sigma_{i}$, respectively, where i∈{1,2,..,m}. Let $\mu_{i}^{\prime }$ denote the mean of the unknown underlying distribution of the control random variable $a_{i}$, and let and $\sigma_{i}^{\prime }$ be the standard deviation. All values of the property $a_{i}$ for the minority class of training data are independent random variable values because they represent independent experiments, and each value obeys a similar potential probability distribution. According to the conclusion of the central limit theorem, when the number of samples n approaches infinity, the distribution of the following random variables under control approaches a normal distribution with zero mean and standard deviation equal to 1.

(5) $$\frac{\mu_{i}-\mu_{i}^{'}}{\sigma_{i}^{'}/\sqrt{n}}\overset{p}{\to }N(0,1)$$

*n* is the number of instances of the minority class. Inspired by (5), given the values $r_{i}$ of the random variables obeying the distribution *N*(0, 1), we obtain the following equation:

(6) $$\mu_{i}^{'}=\mu_{i}-r_{i}\cdot \sigma_{i}^{'}/\sqrt{n}$$

In (6), $\mu_{i}$ is the mean value of the attribute $a_{i}$ for a given training minority class of data, which we consider to be a representative of the original minority class of data. $\mu_{i}^{\prime }$ is the mean value of the attribute $a_{i}$ for the unknown minority class data, which we consider to be a representative of the unknown minority class data. Thus, for any instance and its given value of $a_{i}$, we can generate the synthetic value of this attribute by the following calculation.

(7) $$a_{i}^{'}=a_{i}-r_{i}\cdot \sigma_{i}^{'}/\sqrt{n},i\in \left\{1,2,...,m\right\}$$

In (7), $a_{i}^{\prime }$ is the new value of the property $a_{i}$. $\sigma_{i}^{\prime }$ is unknown, and we approximate it by $\sigma_{i}$ to obtain Equation (8)

(8) $$a_{i}^{'}=a_{i}-r_{i}\cdot \sigma_{i}^{}/\sqrt{n},i\in \left\{1,2,...,m\right\}$$

**Figure 1:**
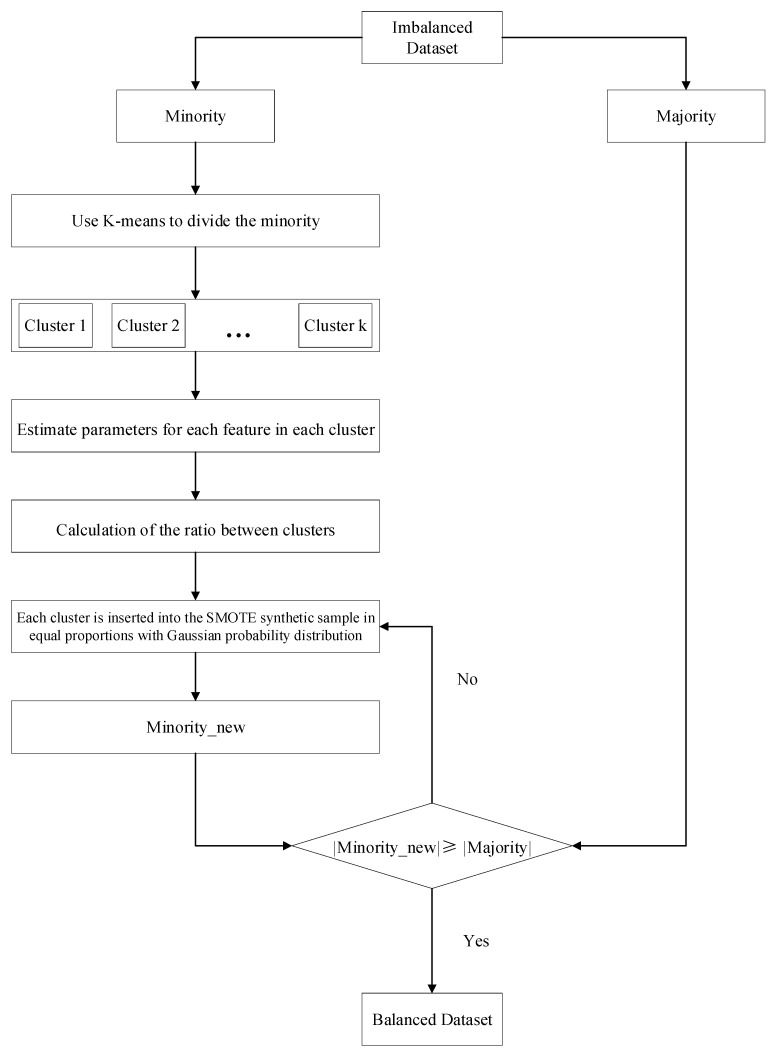
Diagram of the proposed method(KG-SMOTE).

We will refer to (8) as the normal distribution model. The flow chart of its KG-SMOTE algorithm is shown in Figure 1:

The determination of the K-value in this study was carried out using the Elbow Method, which is a commonly employed technique in the K-means algorithm. The Elbow Method evaluates the intra-cluster structure by analyzing the sum squared error (SSE) between each cluster and the samples within it.

The degree of line distortion in the plot represents the spatial variation of the samples within the cluster. A higher degree of distortion indicates a looser cluster structure, while a lower degree of distortion suggests a tighter cluster structure. The Elbow Method aims to identify the point at which the distortion degree starts to exhibit a significantly slower rate of change. This point is typically considered the optimal number of clusters [21].

**Figure 2:**
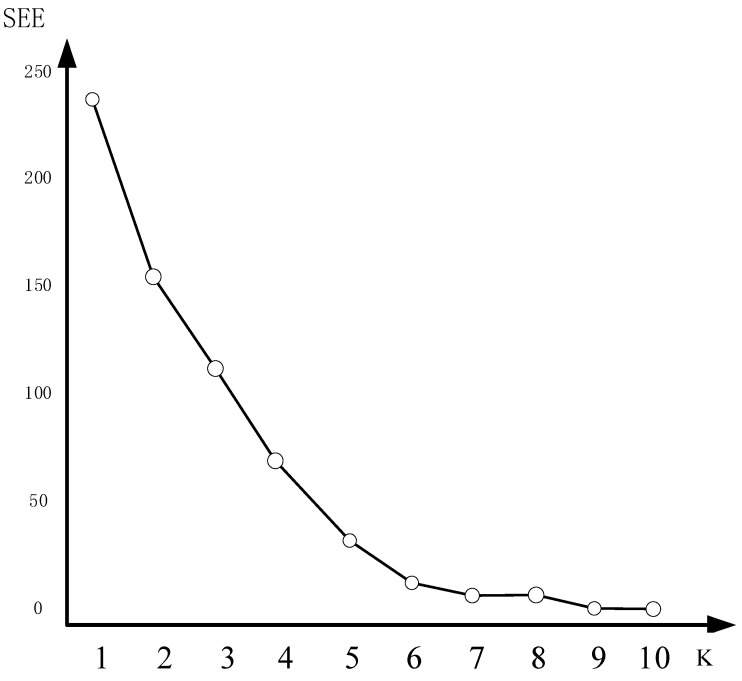
Illustration of the elbow rule effect.

As illustrated in Figure 2, the optimal number of clusters for this dataset was determined to be six, as indicated by the elbow point.

The algorithm employed in this study utilizes a clustering approach to synthesize new samples within clusters. This technique effectively reduces the interference from noise and anomalous samples, enabling the extraction of more valuable information from each cluster. The primary goal is to uncover hidden patterns or groupings within the data.

To generate synthetic data with similar probability distributions as the original minority class data, a Gaussian distribution is applied to each minority cluster. This process involves calculating the normal distribution of all minority-class samples. New samples are then inserted within the range of this calculated distribution. By simulating the distribution characteristics of real data, the generated samples possess higher realism and usability. This approach helps prevent the generation of redundant samples and mitigates the risk of overfitting.

**Figure 3:**
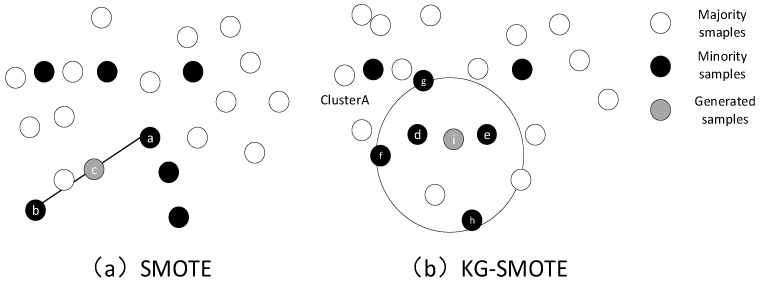
Comparison of synthetic samples between SMOTE and KG-SMOTE improved algorithms.

Furthermore, compared to the traditional SMOTE algorithm, KG-SMOTE does not require the execution of the nearest neighbor algorithm in order to obtain the k-nearest neighbors before creating synthetic samples. This design choice reduces the impact of the parameter settings. Figure 3 provides a visual comparison between the synthetic samples generated by SMOTE and KG-SMOTE. Specifically, let us consider Cluster A as an example. The algorithm calculates the mean and variance of all minority class sample points within Cluster A for each feature. It then employs interpolation to generate a new sample point, denoted as point i, randomly within the Gaussian distribution of the sample points. This new sample point is subsequently added to the minority class sample set.

## 5. Experiments

### 5.1. Dataset

In this paper, we utilized the MBB-IoT dataset [22], which is based on a unique attack scenario. The dataset was developed by a research team from the People’s Public Security University of China. The objective behind constructing this dataset was to simulate real-world IoT environments, where malicious codes were implanted to infiltrate IoT devices. These compromised devices were then utilized to establish a botnet, which was subsequently employed to carry out attacks on internet applications.

The specific experimental scenario involved the creation of an IoT botnet by infecting various devices such as smartphones, webcams, smart speakers, smart gateways, and a Raspberry Pi. The targets of these attacks were servers located outside the local area network (LAN). The network environment consisted of a blend of both benign and botnet traffic. The raw PCAP file obtained from this environment contained a staggering 54 million records. For this study, a subset of the dataset was selected, comprising 1% of the total records, amounting to over 540,000 records. Table 2 presents the imbalance ratios of the attack traffic samples, normal traffic samples, and various attack protocols in the dataset. The ratios range from 1:164 to 1:768, indicating significant imbalances between the different classes. The imbalance ratio, abbreviated as IR, refers to the ratio between the number of instances in the minority class and the number of instances in the majority class in an imbalanced dataset. It is a measure used to quantify the level of class imbalance in a dataset.

Data pre-processing is an essential step in ensuring the quality and usability of a dataset. Raw data often contain irregularities and incompleteness, such as missing values and outliers. Modeling with such data can result in distorted and inaccurate models. Therefore it is necessary to pre-process the raw data before conducting any modeling and analysis.

Data pre-processing typically involves various steps, including data cleaning and data transformation. In this study, the dataset was iterated to identify and handle missing values. Fortunately, no missing values were found in the dataset.

Additionally, features that had values consistently equal to a certain value were removed during the pre-processing stage. The specific features that were removed are listed in Table 3. Following the pre-processing steps, the dataset was left with a total of 77 features.

### 5.2. Experimental Environment and Evaluation Index

The experimental environment and associated hardware are illustrated in Table 4.

Commonly used metrics to evaluate the performance of a classifier include accuracy, precision, recall, F1-score, and AUC (Area Under the Curve). In the case of unbalanced multi-classification problems, accuracy is not a reliable measure of classifier performance. Given the complexity of unbalanced multiclassification, this paper adopts precision, recall, F1-score, and AUC as the evaluation metrics. These metrics are widely used to assess the classification performance of unbalanced data and provide a more comprehensive evaluation of the classifier’s effectiveness [23].

Precision is a commonly used measure to assess the classification accuracy of a classifier for positive class samples. It represents the proportion of samples that are truly positive among all samples predicted to be positive by the classifier. Precision is calculated using the following formula:

(9) $$precision=\frac{TP}{TP+FP}$$

where *TP* denotes the number of true positive samples, *FN* denotes the number of false negative samples, and *FP* denotes the number of false positive samples. By calculating precision, we can evaluate how well a classifier is able to accurately identify positive cases from the total number of samples predicted as positive.

Recall, also known as sensitivity or true positive rate, measures the proportion of samples that are correctly classified as positive out of all samples that are truly positive. It is calculated using the following formula:

(10) $$recall=\frac{TP}{TP+FN}$$

where *TP* represents the number of true positive samples and *FN* represents the number of false negative samples. By calculating the recall, we can assess how well a classifier is able to correctly identify positive cases from the total number of positive instances in the dataset.

The F1-score is a widely used evaluation metric for assessing the performance of classifiers on unbalanced data. It is a harmonic mean of precision and recall, combining both measures into a single value. By considering both precision and recall, the F1-score provides a balanced assessment of a classifier’s performance in scenarios where class distribution is imbalanced.

(11) $$F1-score=\frac{2\cdot precision\cdot recall}{precision+recall}$$

In binary classification problems, the AUC (Area Under the Curve) is a widely used evaluation metric to assess the performance of a classification model. It is calculated by determining the area under the Receiver Operating Characteristic (ROC) curve, which ranges between 0 and 1. The AUC value indicates the classifier’s ability to rank positive and negative cases correctly based on the ROC curve.

An AUC value of 1 suggests that the classifier perfectly distinguishes positive cases from negative cases. Conversely, an AUC value of 0.5 implies that the classifier’s performance is equivalent to random guessing. If the AUC is less than 0.5, it indicates that the classifier’s performance is worse than random guessing.

### 5.3. Experimental Results and Analysis

In this paper, we conducted experiments to compare the performance of classical oversampling algorithms, namely Borderline SMOTE, SMOTE, and K-Means SMOTE, combined with classical decision tree classification algorithms. The MBB-IoT dataset was used for the experimental evaluation, and the results were compared accordingly. Additionally, to validate the effectiveness of oversampling, we included the classification results of the decision tree classification algorithm without any sampling method as a basis of comparison.

The experimental process involved preprocessing the dataset and employing five-fold cross-validation as the evaluation method. We ran the experiments ten times independently and calculated the average results. The experimental findings, as shown in Table 5, Table 6, Table 7 and Table 8, provide precision, recall, F1-score, and AUC values for different algorithms across various types of attack data. The best-performing methods are indicated by bolded entries in the tables.

In Table 5, regarding precision, KG-SMOTE ranks first among the eight attack protocols, demonstrating excellent performance. In the case of the BASHLITE TCP attack type, the selected four attack protocols exhibit outstanding performance, with accuracy exceeding 98%. The difference between them is minimal. This is because the BASHLITE TCP dataset is relatively balanced, and oversampling significantly improves precision. This is due to the oversampling mechanism resulting in an increase in the number of minority class samples in the dataset. By generating more minority class samples through oversampling, the classifier can more fully learn the features of the minority class samples during the training process, thereby improving the correct classification rate of the minority class samples.

However, it is important to note that high precision does not necessarily mean that samples are correctly identified. Precision only considers the ratio of correctly classified positive samples to all samples classified as positive. It does not take into account the number of false negatives, thus not providing a comprehensive reflection of the classifier’s performance.

In highly imbalanced datasets, if the classifier predicts all samples as the majority class, the precision may be high because the majority of samples are correctly classified as the majority class. However, this does not mean that the classifier can correctly identify minority class samples, and the recall rate may be low. Therefore, relying solely on precision to evaluate the performance of the classifier is not comprehensive.

In conclusion, although KG-SMOTE performs well in terms of precision and achieves high accuracy in the BASHLITE TCP attack type, relying solely on precision is not sufficient to comprehensively evaluate the performance of the classifier. It is important to consider other metrics such as recall, F1 score, etc., in order to obtain a more comprehensive and accurate assessment.

In Table 6, it is shown that, in terms of the recall parameter, KG-SMOTE demonstrates excellent performance in seven of the attack types, with an improvement of 9–20% compared to the SMOTE and Borderline SMOTE oversampling methods. When compared to other attack types, the differences between KG-SMOTE and the best algorithm in the BASHLITE TCP, BASHLITE RandHex, and Mirai syn attack types are 0.68%, 1.11%, and 0.88%, respectively, which are relatively small.

The F1 score is a comprehensive performance evaluation metric that combines precision and recall. From Table 7, it can be observed that KG-SMOTE demonstrates leading performance among the eight attack types. Compared to the SMOTE and Borderline SMOTE oversampling methods, KG-SMOTE shows an improvement of at least 20%. Compared to K-means SMOTE, KG-SMOTE achieves an improvement range of 2–6%.

In the attack types where KG-SMOTE did not achieve the top performance, the algorithm only slightly lags behind the best algorithm by 0.4% and 0.38% in the Benign and BASHLITE TCP attack types, respectively. This indicates that the performance gap of KG-SMOTE in these attack types is very small.

According to Table 8, it can be observed that KG-SMOTE outperforms SMOTE, Borderline SMOTE, and K-Means SMOTE in terms of AUC scores in attack protocols with an imbalance ratio of 1:164 or higher. However, KG-SMOTE is not as effective as SMOTE in the BASHLITE TCP attack protocol.

The results depicted in Table 5, Table 6, Table 7 and Table 8 clearly indicate that categorization using raw data (without resampling) yields favorable performance in terms of the precision metric, but it produces poor results when evaluated using the F1 metric. This discrepancy arises because the accuracy metric can be misleading and unreliable when dealing with imbalanced data. This is due to the fact that accuracy solely considers the number of correctly categorized samples, without considering the distribution of instances across the majority and minority categories. In contrast, the F1 metric takes into account the correct classification of samples in both categories, thereby offering a more comprehensive evaluation. These findings underscore the limitations of relying solely on the accuracy metric, as it can lead to misclassification and unreliable outcomes. Conversely, when the KG-SMOTE oversampling method is employed, the classifier’s performance improves significantly in terms of the F1 metric compared to the original data. This improvement can be attributed to the increased number of samples in the minority class, resulting in a more balanced dataset.

When comparing the classification results of unsampled data and SMOTE oversampling, it is apparent that the classification results after employing SMOTE oversampling are significantly lower. This can be attributed to the fact that the synthesized data points are distributed throughout the intervals of multiple classes, giving rise to a large number of noise samples and anomalous samples. This blurs the classification boundaries, making it challenging for the classification algorithm to accurately distinguish between different classes, thereby reducing recognition accuracy.

In contrast, when using the Borderline SMOTE method, synthetic samples are generated along the line connecting minority samples in the feature space. However, in scenarios where there is a substantial disparity between the majority and minority classes, a large number of synthetic data points are required. Consequently, these synthetic samples tend to be concentrated along the same line, resulting in overlapping synthetic samples. This can lead to overgeneralization issues in the model, thereby reducing recognition accuracy.

Furthermore, the Borderline SMOTE algorithm classifies minority class samples into three categories. While this approach may help address the class imbalance in the dataset, it can also result in fewer minority class samples remaining in an already imbalanced dataset. This reduction in minority class samples may cause important features to be overlooked, further contributing to a decrease in recognition accuracy.

**Figure 4:**
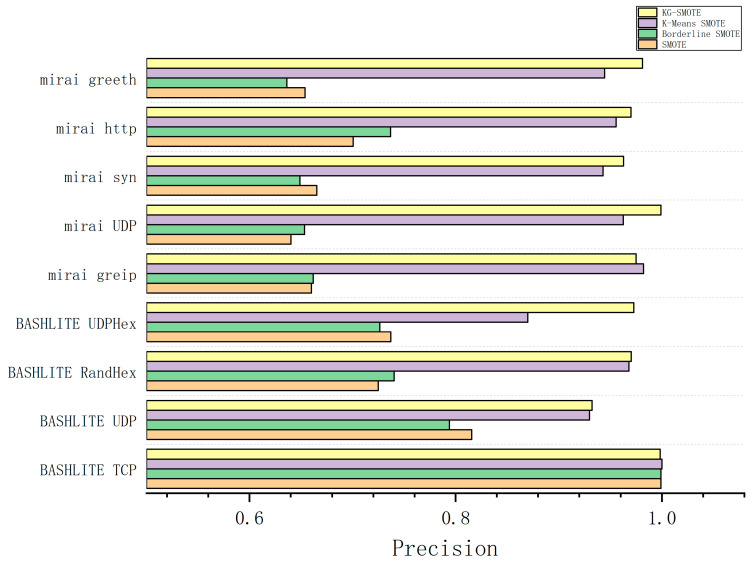
Comparison of precision bars for various oversampling methods.

**Figure 5:**
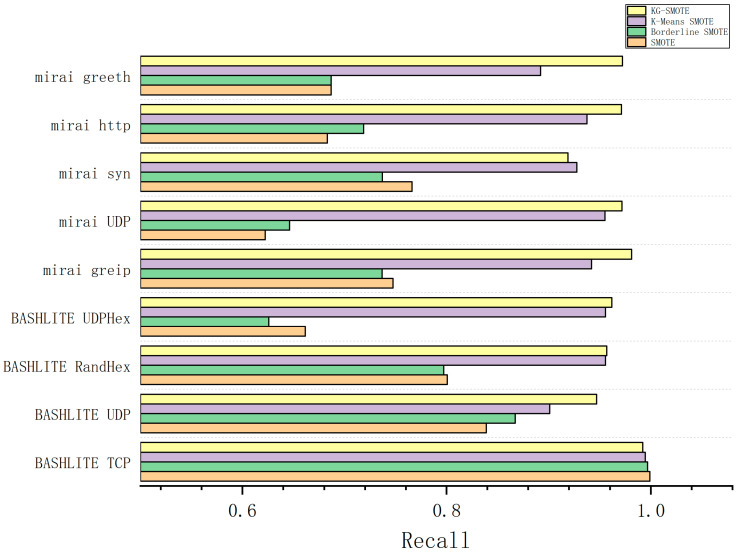
Comparison of recall bars for various oversampling methods.

**Figure 6:**
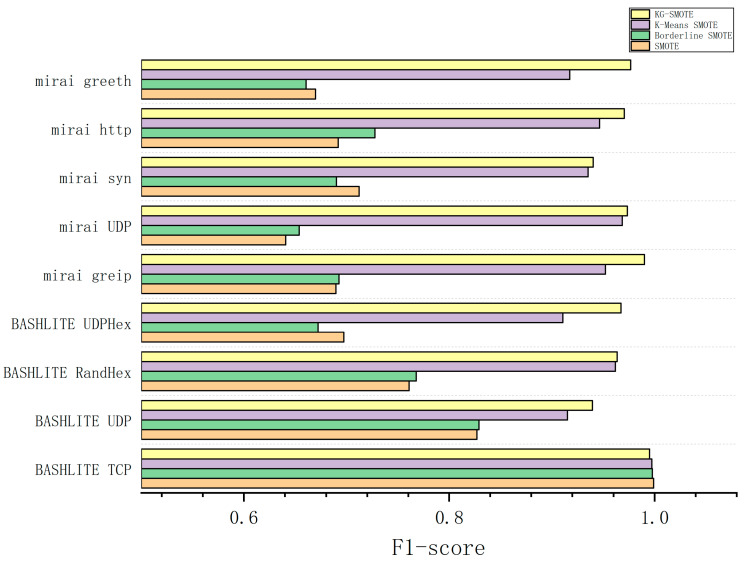
Comparison of F1-score bars for various oversampling methods.

**Figure 7:**
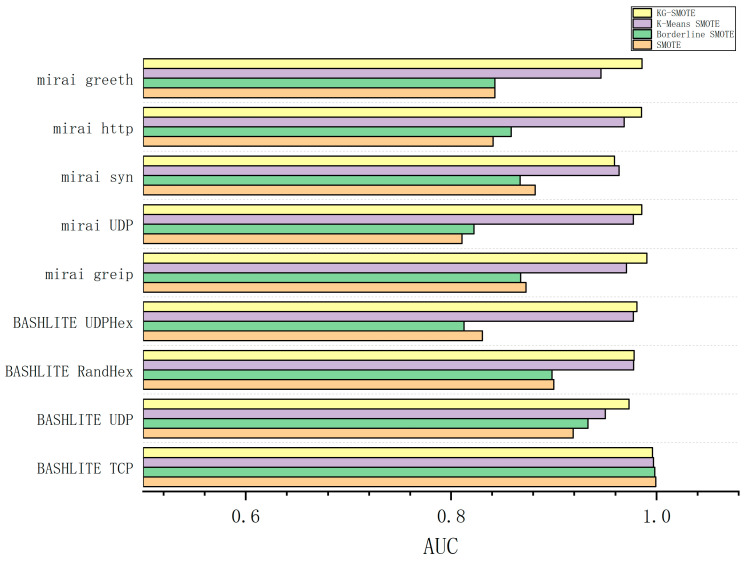
Comparison of AUC bars for various oversampling methods.

From Figure 4, Figure 5, Figure 6 and Figure 7, The KG-SMOTE algorithm offers improvements over both Borderline SMOTE and SMOTE in terms of evaluation metrics such as precision, recall, AUC, and F1 scores across all protocol attacks. This improvement can be attributed to the rational expansion and filtering of the dataset achieved through the selection of points using clustering. Comparing KG-SMOTE with K-Means SMOTE, it is observed that, although K-Means SMOTE takes into account the problem of subclass distribution by dividing the dataset into subclasses, the SMOTE method fails to consider the distribution of samples during the interpolation process. This oversight can lead to overfitting issues, particularly when dealing with datasets that are extremely imbalanced. Notably, KG-SMOTE demonstrates significant improvement in the protocol attacks of Mirai greeth, Mirai HTTP, Mirai greip, Mirai UDP, and BASHLITE UDP. Experimental results indicate that, when synthetic samples follow a Gaussian distribution probability during interpolation, as opposed to randomly generating new samples on the connection line in the feature space between two samples, the detection of attacks becomes more effective and the overall classifier performance improves. This highlights the efficacy of using a Gaussian distribution probability for synthetic sample generation in the context of KG-SMOTE.

From Figure 4, Figure 5, Figure 6 and Figure 7, it can be observed that traditional oversampling methods are not effective in addressing the data imbalance issue in this scenario. In fact, traditional oversampling techniques do not always lead to improved classification performance and can even result in worse outcomes. This is particularly evident in the context of this paper. The paper highlights that the characteristics of IoT traffic and the highly imbalanced datasets in the scenario of DDoS attacks require a tailored oversampling approach. This is why the KG-SMOTE algorithm is proposed to address these specific challenges. By considering the unique characteristics of IoT traffic and the high class imbalance ratio, KG-SMOTE aims to improve the detection effectiveness for DDoS attacks initiated by different virus protocols.

## 6. Conclusions

To accurately simulate real-world IoT attack scenarios, we have utilized the MBB-IoT dataset, which was collected in the context of constructing an IoT botnet by infecting various IoT devices such as smartphones, webcams, smart speakers, smart gateways, and a Raspberry Pi. The attacks were targeted towards servers that were not on the same local area network. Due to the diverse nature of these IoT devices, the dataset exhibits variations in data formats, posing challenges in balancing the dataset.

Unbalanced data present a challenging task for many classification algorithms. Resampling the training data to achieve a more balanced distribution is an effective method to address this issue, independent of the choice of classifier. However, simply replicating instances of the minority class to balance the classes can lead to overfitting, thus reducing the performance of the model on unseen data. On the other hand, techniques that generate artificial samples often tend to produce noisy samples, hindering the inference of class boundaries. Furthermore, most existing oversamplers fail to offset the imbalance within the minority class, which is typically a major problem when classifying imbalanced datasets. In order to effectively assist the classifier through oversampling, it is important to avoid amplifying noise by detecting safe regions in the input space where class regions do not overlap. Additionally, any imbalances within the minority group should be identified and samples generated to achieve a balanced distribution.

In response to the problem of data imbalance in the field of intrusion detection based on the Internet of Things (IoT), this paper proposes a KG-SMOTE algorithm that combines Gaussian probability distribution and the K-Means clustering algorithm. This algorithm utilizes k-means clustering to group the data and concentrate the generation of synthetic samples in the critical regions of the input space. A high proportion of minority observations are used as indicators of safe regions. Oversampling is performed only on the safe clusters, which allows the k-means SMOTE to avoid generating noise. Additionally, the average distance between minority samples within a cluster is used to identify sparse regions. More synthetic samples are generated for the sparse minority groups, which helps alleviate the imbalance within the class. Considering the protocol diversity of IoT devices in DDoS attacks and the heterogeneity of data structures, this approach provides a more effective way of synthesizing samples, thus addressing the class imbalance issue in IoT intrusion detection datasets to some extent. Building upon the K-Means SMOTE oversampling algorithm, new samples are generated using a Gaussian probability distribution. This preserves the sample distribution, increases sample diversity, reduces the overlap ratio of synthetic samples, reduces the risk of classifier overfitting, and improves the detection accuracy of minority class samples.

The main advantages of the KG-SMOTE method are its simplicity, efficiency, and flexibility in generating synthetic samples for the minority class. The KG-SMOTE algorithm performs particularly well for attack types with higher imbalance ratios. For BASHLITE UDPHex (1:768), it outperforms SMOTE, Borderline SMOTE, and K-Means SMOTE in terms of accuracy, precision, recall, F1-score, and AUC. In mirai syn (1:249), it exhibits advantages in accuracy, precision, F1-score, and AUC. However, for BASHLITE TCP (1:164), it does not show any advantage in any of the metrics. The KG-SMOTE algorithm demonstrates strong performance in scenarios with high imbalance. Conversely, it has some limitations in situations with lower imbalance levels. It should be noted that IoT devices have become new actors in launching DDoS attacks, while the targets of these attacks are still in the early stages of internet applications. One future research direction is to further explore and study imbalanced classifiers in this context.

## Figures and Tables

**Table 2:** Number of samples of attack type traffic and normal traffic in the 1% MBB-IoT dataset.

Attack Type	Sample Size	IR
Benign	537,052	
BASHLITE TCP	3268	1:164
BASHLITE UDP	1410	1:380
BASHLITE RandHex	964	1:557
BASHLITE UDPHex	699	1:768
mirai greip	1332	1:403
mirai UDP	1258	1:426
mirai syn	2155	1:249
mirai http	1786	1:300
mirai greeth	1493	1:359

**Table 3:** List of features removed in the pre-treatment stage.

High Categrocal Features	Meaningless Label
bidirectional_ece_packets, src2dst_cwr_packets, bidirectional_cwr_packets, src2dst_ece_packets, if_same_vlan_or_not, dst2src_urg_packets, dst2src_ece_packets, dst2src_cwr_packets, vlan_id	id

**Table 4:** Environmental Configuration.

Experimental Platforms	Environmental Configuration
Operating Systems	Fedora Linux 35 (Workstation Edition)
CPU	Intel(R) Xeon(R) Gold 6346 CPU @ 3.10 GHz
GPU	NVIDIA-SMI 520.61.05
RAM	32 GB
Programming Language	Python3.9.12
torch	2.0.1 + cu118
scikit-learn	1.2.2

**Table 5:** Precision metrics of decision tree classifier based on different oversampling methods.

Index	Category	NR	SMOTE	Borderline SMOTE	K-Means SMOTE	KG-SMOTE
Precision	Benign	0.9983	0.9998	0.9998	0.9985	0.9987
	BASHLITE TCP	0.9853	0.9990	0.9990	1.0000	0.9983
	BASHLITE UDP	0.9169	0.8157	0.7939	0.9299	0.9324
	BASHLITE RandHex	0.9647	0.7247	0.7403	0.9680	0.9704
	BASHLITE UDPHex	0.7633	0.7371	0.7262	0.8699	0.9727
	mirai greip	0.9730	0.6599	0.6618	0.9821	0.9750
	mirai UDP	0.9964	0.6401	0.6533	0.9626	0.9991
	mirai syn	0.9193	0.6653	0.6487	0.9429	0.9629
	mirai http	0.8964	0.7005	0.7368	0.9556	0.9700
	mirai greeth	0.9262	0.6537	0.6362	0.9444	0.9811

**Table 6:** Recall metrics of decision tree classifier based on different oversampling methods.

Index	Category	NR	SMOTE	Borderline SMOTE	K-Means SMOTE	KG-SMOTE
Recall	Benign	0.9985	0.9988	0.9987	0.9991	0.9991
	BASHLITE TCP	0.9904	0.9990	0.9969	0.9946	0.9922
	BASHLITE UDP	0.8203	0.8389	0.8673	0.9009	0.9469
	BASHLITE RandHex	0.9679	0.8007	0.7972	0.9556	0.9568
	BASHLITE UDPHex	0.8208	0.6615	0.6256	0.9556	0.9619
	mirai udp	0.9295	0.7473	0.7367	0.9419	0.9812
	mirai greip	0.9654	0.6223	0.6461	0.9552	0.9718
	mirai syn	0.8652	0.7661	0.7370	0.9276	0.9188
	mirai http	0.9565	0.6832	0.7186	0.9374	0.9712
	mirai greeth	0.9498	0.6867	0.6867	0.8921	0.9723

**Table 7:** F1-score metrics of decision tree classifier based on different oversampling methods.

Index	Category	NR	SMOTE	Borderline SMOTE	K-Means SMOTE	KG-SMOTE
F1-score	Benign	0.9985	0.9993	0.9992	0.9988	0.9989
	BASHLITE TCP	0.8878	0.9990	0.9979	0.9973	0.9952
	BASHLITE UDP	0.7659	0.8271	0.8290	0.9151	0.9396
	BASHLITE RandHex	0.8663	0.7608	0.7677	0.9618	0.9635
	BASHLITE UDPHex	0.7910	0.6973	0.6722	0.9107	0.9673
	mirai udp	0.8618	0.6896	0.6925	0.9521	0.9901
	mirai greip	0.8692	0.6406	0.6538	0.9685	0.9734
	mirai syn	0.8914	0.7122	0.6901	0.9352	0.9403
	mirai http	0.8254	0.6918	0.7276	0.9464	0.9706
	mirai greeth	0.8378	0.6698	0.6605	0.9175	0.9767

**Table 8:** AUC metrics of decision tree classifier based on different oversampling methods.

Index	Category	NR	SMOTE	Borderline SMOTE	K-Means SMOTE	KG-SMOTE
AUC	Benign	0.9983	0.9975	0.9967	0.9808	0.9830
	BASHLITE TCP	0.9251	0.9994	0.9984	0.9972	0.9960
	BASHLITE UDP	0.9100	0.9190	0.9332	0.9503	0.9733
	BASHLITE RandHex	0.9838	0.8999	0.8982	0.9777	0.9783
	BASHLITE UDPHex	0.9101	0.8305	0.8126	0.9776	0.9809
	mirai udp	0.9647	0.8729	0.8677	0.9708	0.9906
	mirai greip	0.9826	0.8105	0.8224	0.9775	0.9858
	mirai syn	0.9323	0.8819	0.8673	0.9636	0.9592
	mirai http	0.9779	0.8408	0.8586	0.9686	0.9855
	mirai greeth	0.9747	0.8427	0.8426	0.9459	0.9860

## Data Availability

Not applicable.
